# Exploring the Relationship Between Gene Expression and Low-Frequency Somatic Mutations in *Arabidopsis* with Duplex Sequencing

**DOI:** 10.1093/gbe/evae213

**Published:** 2024-10-04

**Authors:** Gus Waneka, Braden Pate, J Grey Monroe, Daniel B Sloan

**Affiliations:** Department of Biology, Colorado State University, Fort Collins, CO, USA; Department of Biology, Colorado State University, Fort Collins, CO, USA; Department of Plant Sciences, University of California, Davis, Davis, CA, USA; Department of Biology, Colorado State University, Fort Collins, CO, USA

**Keywords:** environment-specific fitness effects, somatic mutation, duplex sequencing, mismatch repair, base excision repair, mutation variation

## Abstract

Intragenomic mutation rates can vary dramatically due to transcription-associated mutagenesis or transcription-coupled repair, which vary based on local epigenomic modifications that are nonuniformly distributed across genomes. One feature associated with decreased mutation is higher expression level, which depends on environmental cues. To understand the magnitude of expression-dependent mutation rate variation, we perturbed expression through a heat treatment in *Arabidopsis thaliana*. We quantified gene expression to identify differentially expressed genes, which we then targeted for mutation detection using duplex sequencing. This approach provided a highly accurate measurement of the frequency of rare somatic mutations in vegetative plant tissues, which has been a recent source of uncertainty. Somatic mutations in plants may be useful for understanding drivers of DNA damage and repair in the germline since plants experience late germline segregation and both somatic and germline cells share common repair machinery. We included mutant lines lacking mismatch repair (MMR) and base excision repair (BER) capabilities to understand how repair mechanisms may drive biased mutation accumulation. We found wild-type (WT) and BER mutant mutation frequencies to be very low (mean variant frequency 1.8 × 10^−8^ and 2.6 × 10^−8^, respectively), while MMR mutant frequencies were significantly elevated (1.13 × 10^−6^). Interestingly, in the MMR mutant lines, there was no difference in the somatic mutation frequencies between temperature treatments or between highly versus lowly expressed genes. The extremely low somatic variant frequencies in WT plants indicate that larger datasets will be needed to address fundamental evolutionary questions about whether environmental change leads to gene-specific changes in mutation rate.

**Significance**

Accurately measuring mutations in plants grown under different environments is important for understanding the determinants of mutation rate variation across a genome. Given the low rate of de novo mutation in plant germlines, such measurements can take years to obtain, hindering tests of mutation accumulation under varying environmental conditions. We implemented highly accurate duplex sequencing to study somatic mutations in plants grown in two different temperatures. In contrast to plants with deficiencies in DNA mismatch repair machinery, we found extremely low mutation frequencies in wild-type plants. These findings help resolve recent uncertainties about the somatic mutation rate in plant tissues and indicate that larger datasets will be necessary to understand the interaction between mutation and environment in plant genomes.

## Introduction

In plant nuclear genomes, coding sequences incur fewer mutations than noncoding sequences, and essential genes accumulate fewer mutations than nonessential genes (Ossowski et al. 2010; Weng et al. 2019; Monroe et al. 2022; Quiroz et al. 2023; Monroe et al. 2023a; Monroe et al. 2023b). Such decreased local mutation rates likely derive from increased protection of important sequences by DNA repair machinery (Quiroz et al. 2024; Monroe 2023). For example, it has long been established that transcription-coupled nucleotide excision repair (TC-NER) leads to increased protection of the transcribed strand of genes via recognition of RNA polymerases which become stalled at damaged sites as a cue for recruitment of NER machinery (van Gool et al. 1997; Selby et al. 2023). In plants exposed to UV, the prevalence of active NER sites appears to be correlated with expression level (Oztas et al. 2018). In addition it has been recently proposed that mismatch repair (MMR) in plants may provide increased protection to highly expressed genes through the recognition of histone modifications associated with increased expression by the mismatch recognition complex (Quiroz et al. 2024).

Increased repair of highly expressed genes in plants may result in environment-specific mutation profiles, which has interesting implications for plant adaptation and evolution (Zhang 2023). Alternatively, transcription-associated mutagenesis may occur due to increased DNA damage associated with exposure of single-stranded DNA to mutagens and can potentially overpower the increased protection of actively transcribed genes (Kim et al. 2007; Jinks-Robertson and Bhagwat 2014; Seplyarskiy et al. 2023). However, the magnitude and relative importance of expression-dependent local mutation rate variation are not completely understood.

A challenge associated with addressing how local mutation rates vary with environment is the difficulty of measuring mutations in experimental settings. Historical estimates of mutation relied on comparisons of synonymous substitutions between populations or species. Because these substitutions do not result in a change in amino acid, they are expected to experience minimal selection and thus approximate mutational input, though in reality synonymous sites do experience selection due to codon usage bias (Grantham et al. 1980; Hershberg and Petrov 2008) and other mechanisms (Bailey et al. 2021). It is inherently difficult to measure mutation rates more directly in large multicellular organisms because their long generations require many individuals and/or large amounts of time for sufficient mutations to occur, making methods such as mutation accumulation lines and parent–offspring trio sequencing (Lynch et al. 2016; Tatsumoto et al. 2017) expensive and time-consuming.

An alternative and potentially complementary approach to mutation accumulation and trio sequencing studies is to detect the mutations that accumulate in an organism's somatic tissues (Gundry and Vijg 2012; Moore et al. 2021; Monroe et al. 2022; Quiroz et al. 2023; Satake et al. 2023; Schmitt et al. 2024; Staunton et al. 2023; Goel et al. 2024). This approach benefits from the fact that many more cell lineages can be tracked than just the germline. Though selection acts on inherited mutations, germline and somatic cells tend to display similar spectra and relative rates due to shared DNA replication and repair machinery (Beichman et al. 2024). In contrast to metazoans, where germline and somatic mutations are clearly distinct (Zhang and Vijg 2018), plant somatic and germline mutations are often indistinguishable due to the late differentiation of germline cells from meristematic cells after vegetative growth (Watson et al. 2016). As a result, a somatic mutation that arises in a single plant branch may also contribute to the germline via reproductive organs on the branch (Quiroz et al. 2023; Goel et al. 2024).

Inclusion of somatic (vegetative) mutations in recent *Arabidopsis* studies led to the identification of thousands of mutations, which increased power to test for relationships between local mutation rates and various sequence features, such as GC content, DNA methylation, histone modifications, and expression level (Monroe et al. 2022). However, this approach appears to have been inaccurate because low-frequency somatic variants can be difficult to distinguish from sequencing errors, and reanalysis of the somatic mutation calls showed that many of the putative mutations arose from technical artifacts (Liu and Zhang 2022; Wang et al. 2023; Monroe et al. 2023a; Monroe et al. 2023b). A follow-up analysis that increased the stringency of somatic mutation calls to obtain a new set of high-confidence somatic mutations showed that local mutation rates are lower in genes than in intergenic sequences and within genes mutation rates tend to correlate negatively with expression (Monroe et al. 2023a). The same patterns were observed in a parallel analysis of germline mutations, highlighting the similarities in distribution between somatic and germline mutations in plants (Monroe et al. 2022; Monroe et al. 2023a). Still, the actual frequency of somatic mutations in vegetative plant tissue remains an open question.

Measurements of low-frequency somatic mutations can be obtained using a high-fidelity sequencing technology to distinguish mutational signal from noise (Sloan et al. 2018). For example, duplex sequencing is an Illumina-based method in which unique molecular identifiers (UMIs) are included in adaptors and attached to both ends of DNA fragments before library amplification (Schmitt et al. 2012; Kennedy et al. 2014). After sequencing, the UMIs are used to cluster families of reads that originated from each strand of a given DNA fragment so that a double-stranded consensus sequence can be created that is virtually error free (<5 × 10^−8^ errors per base pair; Kennedy et al. 2014).

Our goal in this study was to test if local mutation rate variation across plant genomes depends on environmentally determined gene expression levels. We also wanted to determine whether low-frequency somatic mutations in plant tissues could provide a robust signal for addressing this type of question. Therefore, we perturbed gene expression by growing *Arabidopsis* under different temperatures. We identified differentially expressed (DE) genes with RNA-Seq, which we then targeted for low-frequency somatic mutation detection using duplex sequencing coupled with hybrid capture. We included mutant lines *msh2* and *ung*, which, respectively, lack MMR and base excision repair (BER) capabilities, in order to understand how repair mechanisms may drive biased mutation accumulation (Cordoba-Canero et al. 2010; Belfield et al. 2018). We also included *hsp70-16* mutant lines, which are deficient for a key heat shock protein, as a means to endogenously manipulate gene expression and potentially interact with our temperature treatment (Ran et al. 2020). As expected, we found significant increases in variant frequencies in the MMR-deficient lines. In wild-type (WT) lines and other mutant lines, measured mutation frequencies were too low to quantify relationships between mutation rates and environment-specific gene expression levels. Therefore, our results support the conclusion that earlier estimates of somatic variant frequencies were inflated (Wang et al. 2023; Monroe et al. 2023a) and indicate that much larger datasets will be needed to test for environment-specific changes in mutation biases.

## Results

To test if environment-specific changes in gene expression impact mutation, we performed mutation detection on a targeted set of *Arabidopsis* genes that were DE in plants grown at 20 °C versus 30 °C. We first generated and analyzed RNA-seq data to identify genes in six categories: (i) increased expression at 30 °C compared to 20 °C in WT plants, (ii) increased expression at 20 °C compared to 30 °C in WT plants, (iii) constitutively high expression in WT plants at both 20 °C and 30 °C, (iv) constitutively low expression in WT plants at both 20 °C and 30 °C, (v) genes that had increased expression at 30 °C versus 20 °C in WT plants (like category i) and also had an interaction between WT and *hsp70-16*, and (vi) genes that had increased expression at 30 °C versus 20 °C in WT plants (like category ii) and also had an interaction between WT and *hsp70-16.* The sequences of the DE genes were used to create a custom probe-set for hybrid capture of duplex sequencing libraries (supplementary table S1, Supplementary Material online).

For genes to be placed in category i or ii, we required that they have a minimum normalized coverage of at least five reads, a corrected *P*-value of <0.05, and a log2 fold-change of >2 (supplementary table S1, Supplementary Material online). Under these criteria, we found that 615 genes had significantly increased expression at 30 °C (category i), while 332 genes had significantly increased expression at 20 °C (category ii; supplementary fig. S1, Supplementary Material online). From each of category i and ii, we selected the 100 genes with the largest log2 fold-change to be included in the custom probe-set (supplementary fig. S1 and table S1, Supplementary Material online).

Duplex sequencing coverage of the genes and 250 bp of flanking sequence in the probe-set ranged from 74.7× to 109.4× (supplementary fig. S2, Supplementary Material online), and the average probe-set coverage across all libraries was 193.1-fold higher than the genome background. In total, we obtained 1.89 Gb of duplex sequencing coverage of our region of interest across the 24 libraries (supplementary table S2, Supplementary Material online).

We then looked for the presence of single-nucleotide variants (SNVs) and short insertions/deletions (indels) within the 339 genes covered in the probe-set. Mutant alleles already present in the parents of the assayed sets of full-sib plants have the potential to bias estimates of de novo mutation frequencies but should be readily identifiable. For a homozygous parent, they would be present in all duplex sequencing reads of all the replicates of a given genotype. For a heterozygous parent, they would segregate in a 1:2:1 Mendelian ratio and account for roughly 50% of the reads for all replicates of a given genotype (as each replicate represents a pool of five sibling plants). We identified just three apparent fixed SNVs (supplementary table S3, Supplementary Material online), which were removed for downstream analyses. In contrast, we identified 41 fixed indels, over half of which were in the *msh2* background (supplementary table S4, Supplementary Material online). One gene (AT5G39190) had five sites that appeared to be segregating SNVs in all 24 replicates. We suspected this might be caused by a cryptic gene duplication that was not captured in the TAIR 10.2 reference genome (Jaegle et al. 2023). Indeed, when we realigned the reads to the improved Col-CC genome (Reiser et al. 2024), the mutation calls in AT5G39190 were absent. As such, reads mapping to AT5G39190 were disregarded in downstream analyses. The rest of the SNVs we identified were unique to each replicate and all were present at a frequency of no more than 17.64% (the average variant frequency across all mutations was 2.27%), suggesting that these are low-frequency somatic variants that arose during the experiment and were present in a subset of the sampled vegetative tissue.

**Fig. 1.**
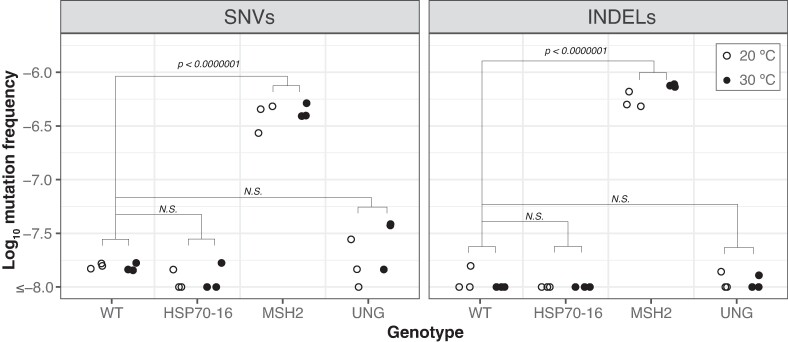
Mutation frequencies in WT versus mutant lines at 20 and 30 °C. Log_10_ mutation frequencies for SNVs and indels calculated as the number of events (SNVs or indels) divided by the duplex sequencing coverage of the probe-set. A floor of 1 × 10^−8^ was applied to the *y*-axis for data visualization. *P-values* are from a Tukey's test on a two-way ANOVA performed in R with the emmeans package (version 1; Lenth et al. 2021).

**Fig. 2.**
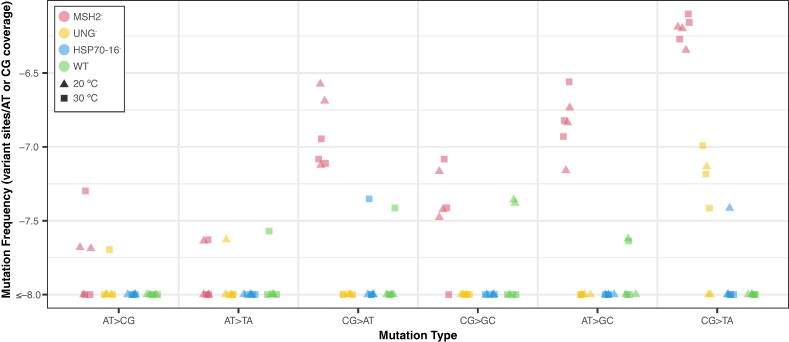
Mutation spectrum for WT and mutant plants at 20 and 30 °C. Log_10_ mutation frequencies for different types of SNVs were calculated as the number of events divided by the nucleotide-specific duplex sequencing coverage of the probe-set. A floor of 2.5 × 10^−8^ was applied to the *y*-axis for data visualization.

Among the six WT biological replicates, we detected a single indel and just six SNVs, one in each replicate (Fig. 1). As such, there was very limited statistical power to test for the effects of temperature or expression level on mutation frequency in WT plants. Similarly, we detected few or no SNVs and indels in the *hsp70-16* and the *ung* mutant lines (Fig. 1; supplementary Files S1 and S2, Supplementary Material online). In contrast, variant frequencies were significantly elevated in the *msh2* mutant lines (compared to WT plants), where we detected 271 indels and 180 SNVs (Fig. 1; two-way ANOVA with Tukey's test, *P* < 0.0001). The mutations in the *msh2* lines were distributed relatively evenly across the temperature treatments, as we found that temperature did not influence either SNV or indel frequency (Fig. 1; two-way ANOVA, *P* = 0.99). In the *msh2* lines, deletions were 8.5-fold more common than insertions (supplementary table S5, Supplementary Material online; two-way ANOVA, *P* < 0.0001). We observed significant differences among SNV classes in *msh2* SNV spectrum (Fig. 2; two-way ANOVA, *P* < 0.0001), which was dominated by CG → TA transitions. The next most common types of substitutions were AT → GC transitions and CG → AT transversions. We compared the *msh2* mutation frequencies in the constitutively lowly expressed (group 3 in supplementary table S1, Supplementary Material online) versus constitutively highly expressed (group 4 in supplementary table S1, Supplementary Material online) genes and found no significant differences (paired *t*-test; supplementary table S6, Supplementary Material online), though we did observe a trend toward higher indel frequencies in constitutively highly expressed genes at 30 °C. We did not analyze the SNV spectra or indel bias in WT, *ung*, *or hsp70-16* lines because the small number of sampled mutations precluded a statistically meaningful comparison.

## Discussion

In this study, we took a novel approach to studying plant mutation by utilizing high-fidelity duplex sequencing to measure low-frequency somatic variants in a targeted region of the *A. thaliana* nuclear genome. Variants in unopened floral bud tissue of WT plants were present at very low frequencies (Fig. 1), which were near the detection threshold of duplex sequencing (Kennedy et al. 2014; Wu et al. 2020). Although we did not have enough power to address our prediction that increases in gene expression would correlate with decreases in mutation rates in WT plants, the results are nonetheless of interest given recent debates about the frequency of somatic mutations in plant tissues (Liu and Zhang 2022; Monroe et al. 2022; Wang et al. 2023; Monroe et al. 2023a; Monroe et al. 2023b). Our results support the conclusion that the high error rate of Illumina short-read sequencing makes it difficult to reliably discern sequencing errors from extremely rare WT somatic mutations. That said, we are skeptical of directly comparing the variant frequencies we measured in unopened floral buds with those obtained in differentiated leaves (Monroe et al. 2022; Monroe et al. 2023a) given recent evidence showing substantial variation in somatic mutation rates depending on plant tissue (Goel et al. 2024).

We also surveyed variant frequencies in *ung* mutant plants and did not observe a difference between WT and *ung* lines. Given that *ung* plants have previously been shown to accumulate more uracil in DNA (presumably to the loss of base excision repair activity on deaminated cytosines) than WT plants (Cordoba-Canero et al. 2010), we interpret the lack of a difference between WT and *ung* lines as evidence that actual WT mutation frequencies may be below the detection threshold of duplex sequencing. However, it is also possible that the similarly low mutation rates in WT and *ung* reflect the lack of a true biological difference, which may be possible if redundant pathways exist that prevent uracils in DNA from becoming CG → TA transitions.

In contrast, we found significantly elevated variant frequencies in *msh2* mutants compared to WT lines (Fig. 1). MSH2 is known to function in MMR and mutation accumulation experiments with *msh2* mutant lines have established that the germline SNV rate is 132 to 204-fold greater than the WT SNV rate (Ossowski et al. 2010; Jiang et al. 2014; Belfield et al. 2018). Here, we found that the average *msh2* SNV frequency was 27-fold greater than the average WT SNV frequency (Fig. 1). Though somatic variant frequencies measured with duplex sequencing are not directly comparable to germline mutation rates assayed with mutation accumulation experiments, the smaller magnitude of the difference between *msh2* versus WT in our dataset may be interpreted as further evidence that the actual WT variant frequency is beneath the detection threshold of duplex sequencing. Alternatively, the smaller difference between WT and *msh2* reported here could be evidence that MMR is particularly important for buffering against mutation in germline plant tissues, which is supported by elevated expression of *MSH2* and other mismatch repair genes in meristematic tissues (Klepikova et al. 2016).

Variant frequencies in the *msh2* mutant lines showed no significant difference in plants grown at 20 °C versus 30 °C. This finding contrasts with a recent mutation accumulation study that found elevated germline mutation rates in WT plants grown at 29 °C compared to those grown at 23 °C (Belfield et al. 2021) and another study that documented increases at 28 °C and 32 °C compared to 23 °C (Lu et al. 2021). One potential explanation of this result is that heat stress may be mutagenic in WT plants *because* it impairs MMR since in the absence of MMR there is no apparent heat effect. However, this interpretation would be at odds with the fact that the genome-wide distribution of mutations in the heat-stressed plants mirrors the distribution of WT plants grown at standard temperature, not of mismatch repair mutants (see Figure 3 of Belfield et al. 2021). The duplex sequencing variant frequencies in the ­*msh2* mutant lines also did not vary significantly between lowly expressed versus highly expressed genes at either 20 or 30 °C (Fig. 1). This result is consistent with the model that MMR provides special protection to actively transcribed genes (Belfield et al. 2018; Huang et al. 2018; Huang and Li 2018). However, we present this interpretation cautiously in the absence of WT data to test for an impact of expression when MMR is functional.

In summary, we took a novel approach to studying plant mutations by using duplex sequencing and hybrid capture to obtain a highly accurate snapshot of somatic variants in targeted regions of the *A. thaliana* genome. We designed our experiment to test if environmental conditions alter mutation rates in a gene-specific fashion. However, the low rate of mutations in WT plants prevented testing for how expression levels impact mutation rates. Nonetheless, the link between increased expression and decreased mutation in plants is well-documented (Oztas et al. 2018; Monroe et al. 2022; Quiroz et al. 2023), as is the fact that gene expression is environmentally determined (Richards et al. 2012), so by logical extension environmental conditions must drive mutation rates and related fitness consequences. However, whether the magnitude of such an effect is biologically meaningful in shaping mutation and evolution remains an important, unanswered question. Though mutation accumulation and parent–offspring sequencing are time- and resource-intensive experiments, they are both increasingly feasible due to continued declines in the cost of DNA sequencing (Ossowski et al. 2010; Weng et al. 2019; Monroe et al. 2022). Conducting such experiments under contrasting environments (Jiang et al. 2014; Belfield et al. 2021; Lu et al. 2021) to measure the correlation between expression and mutation seems to be the key to understanding how environments impact the types of mutations that organisms accumulate.

## Materials and Methods

All plants were grown in environmentally controlled growth chambers (75% humidity) under a long-day photoperiod (16-h light, 8-h dark) with irradiance of 185 µmol m^−2^ s^−1^ at constant temperatures (either 20 or 30 °C, as specified below). Prior to planting, seeds were stratified for 5 days in sterile ddH20. *A. thaliana* ecotype Col-0 was used as the WT line. Existing mutant lines were obtained from the Arabidopsis Biological Resource Center (supplementary table S7, Supplementary Material online), and seedlings were screened with allele-specific PCR markers to identify plants that were homozygous for the mutant alleles used in this study (*msh2*, *ung, hsp70-16;*supplementary table S8, Supplementary Material online).

Sibling plants (roughly 35 for each genotype and each temperature treatment) were planted in 2.5-in. pots. Both temperature treatments were initiated in chambers (Conviron models PGR15 [20 °C] and PGCFLEX [30 °C]) at 20 °C because elevated ambient temperatures (30 °C) can inhibit seed germination (Silva-Correia et al. 2014). After 5 days, the temperature was turned up for the 30 °C treatment and kept at 20 °C for the other treatment. When the plants had reached stage 6.5 of development (where ∼50% of flowers have opened) (Boyes et al. 2001), we performed DNA and RNA extractions on unopened floral buds from laterally branching florets. The 30 °C plants reached developmental stage 6.5 at 31 days, while the 20 °C plants reached developmental stage 6.5 at 41 days, consistent with faster plant development at elevated ambient temperatures (Silva-Correia et al. 2014).

For the RNA extractions, plant material was collected from the unopened floral buds of three laterally branching florets from three WT and three *hsp70-16* plants in each temperature treatment. The harvested tissues were immediately placed into liquid nitrogen and homogenized for 10 s at 30 beats/s with the Qiagen TissueLyser, before being processed with the Qiagen RNeasy Plant Mini Kit, according to manufacturer's instructions. The RNA samples were then sent to Novogene and RNA-Seq libraries were made using the NEBNext Ultra II Directional RNA Library Prep Kit with the NEBNext Poly(A) mRNA Magnetic Isolation Module. The RNA-Seq libraries were sequenced on a NovaSeq 6000 using the PE150 strategy to generate 29 to 54 million read pairs per library (see supplementary table S9, Supplementary Material online).

Tissue was harvested for DNA sequencing and mutation detection at the same time as the tissue for RNA extraction, from siblings of the plants used for RNA extraction. For each replicate in the DNA extractions, plant material was pooled from five siblings from the unopened floral buds of three laterally branching florets from five plants per each replicate, with three replicates per genotype (WT, *hsp70-16, msh2, ung*) per temperature treatment. The floret tissue was homogenized for 10 s at 30 beats/s with the Qiagen TissueLyser, before being processed with the DNeasy Plant Mini Kit from Qiagen.

The RNA-Seq reads were analyzed to detect DE genes at 20 °C versus 30 °C. First, the adaptors were removed with Cutadapt version 4.0 with Python 3.9.16 (Martin 2011). Then, the reads were mapped to the TAIR10.2 reference genome with HISAT2 (version 2.2.1; Kim et al. 2019). Read counts were generated with HTSeq-count version 2.0.2 (Anders et al. 2014) and normalized with the DESeq2 median of ratios method (Love et al. 2014). Finally, DESeq2 models were implemented to identify genes that were differentially expressed or constitutively highly or lowly expressed.

We created a custom probe-set to enrich the sequences of DE genes via hybrid capture so that we could perform mutation detection with duplex sequencing. We sent the sequences of 400 DE genes (plus 250 nt of flanking sequence on the end of each gene) to the probe design team at Arbor Bioscience, which flagged 61 of the genes as unsuitable for hybrid capture because they were >25% soft-masked for repeats in a BLAST search against the Arbor Biosciences eudicot database. The remaining 339 genes (listed in supplementary file S2, Supplementary Material online) and flanking sequences spanned a total length of 855,123 nt. Sets of 80 nt probes were 2× tiled across the target sequence at approximately every 40 nt. The probes were biotinylated so that probe-bound library molecules can be captured with streptavidin-coated magnetic beads.

We created duplex sequencing libraries from the 24 DNA samples (3 replicates × 4 genotypes × 2 temperature treatments), following our previously described library preparation protocols (Wu et al. 2020; Waneka et al. 2021), except that in this case the amount of input DNA was increased to 500 ng because the target sequence comprises a small fraction (<1%) of the total cellular DNA sample. Once DNA samples had been fragmented via ultrasonication, end-repaired, A-tailed, adaptor-ligated, and treated with a cocktail of damage removal enzymes (Wu et al. 2020), we amplified 0.73 ng of DNA (per reaction) for 13 PCR cycles with New England Biolabs Q5 high-fidelity polymerase and dual-indexed primers. We then created three pools by combining 350 ng of each amplified library as the Arbor Biosciences hybrid-capture reactions have enough capacity for eight libraries in each pool. We performed the overnight hybrid-capture reaction at 65 °C, according to the manufacturer's instructions (Arbor Biosciences MyBaits Kit Manual v. 5.02). We assessed enrichment efficiency and library concentrations through qPCR (as previously described; Waneka et al. 2021) before amplifying the enriched pools for an additional nine cycles to obtain sufficient library amounts for sequencing.

Duplex sequencing libraries were sequenced with PE150 reads on an Illumina NovaSeq 6000 S4 Lane (Novogene) to generate 87 to 123 million read pairs per library (supplementary table S10, Supplementary Material online). Processing of the duplex sequencing reads was performed with our previously described pipeline (Wu et al. 2020), which trimmed adaptor sequences, created duplex consensus sequences based on the presence of shared barcodes, mapped the consensus sequences to the entire TAIR10.2 reference genome. Each duplex consensus sequences is composed of at least six Illumina reads (at least three originating from each strand of a DNA fragment). Alignment files were then parsed to identify duplex consensus sequences that contain SNVs and short indels. Since duplex sequencing is highly accurate (<5 × 10^−8^ errors per base pair; Kennedy et al. 2014), we require just a single duplex consensus to support a putative mutation. Comparisons of coverage in the probe-set versus outside the probe-set were performed with Samtools version 1.6 (Li et al. 2009). For variant frequency calculations, we excluded the first or last 10 bps of a read because we have previously identified elevated mutation frequencies at read ends (Wu et al. 2020).

## Supplementary Material

evae213_Supplementary_Data

## Data Availability

The raw reads are available via the NCBI Sequence Read Archive under accessions SRR27564102-SRR27564113 (RNA-seq libraries) and SRR27693810-SRR27693833 (duplex sequencing libraries). Duplex sequencing datasets were processed with a previously published pipeline (https://github.com/dbsloan/duplexseq) (Wu et al. 2020).
