# Supplementary material for: Exploring the Relationship Between Gene Expression and Low-Frequency Somatic Mutations in Arabidopsis with Duplex Sequencing
Source: Genome Biol Evol. 2024 Oct 4;16(10):evae213. doi: 10.1093/gbe/evae213 (PMC11489876; doi:10.1093/gbe/evae213)
Supplement: evae213_Supplementary_Data [file evae213_supplementary_data.zip › athal_mut_waneka_GBE.supp_mat.fig.240907.pdf]

SUPPLEMENTARY FIGURES

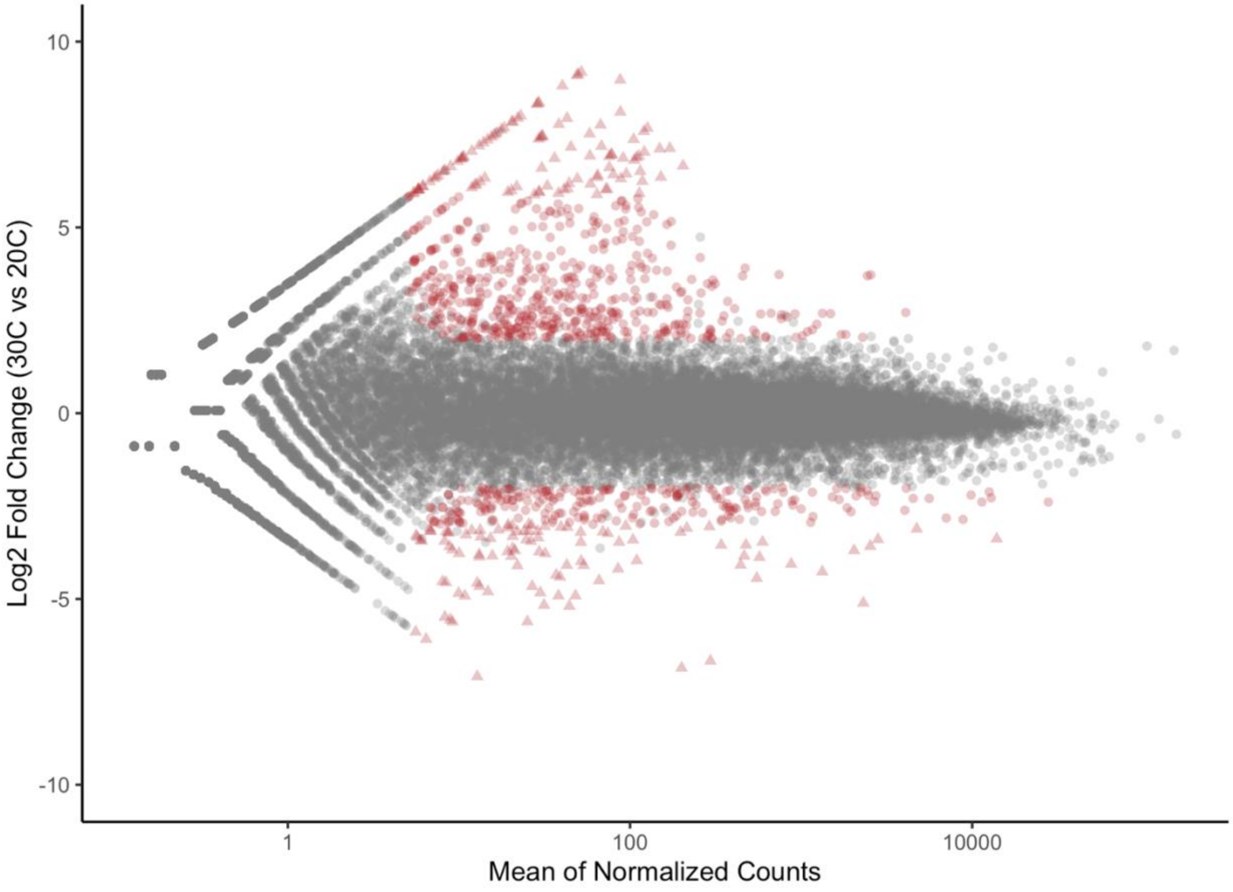

Figure S1. MA plot depicting genes with differential expression in WT plants grown at 20°C or 30°C. To be considered significantly differentially expressed and to be placed in category 1 or 2 (see results), genes were required to have normalized counts of at least 5, have corrected p-values below 0.05, and have log2 fold changes above 2 (increased expression at 30°C; category 1) or below negative two (increased expression at 20°C; category 2). The 615 category 1 genes and the 332 category 2 genes that meet our criteria are plotted in red. We selected 100 category 1 and 100 category 2 genes with the largest log2 fold changes to be included in our probe-set, and these genes are depicted as red triangles in the MA plot.

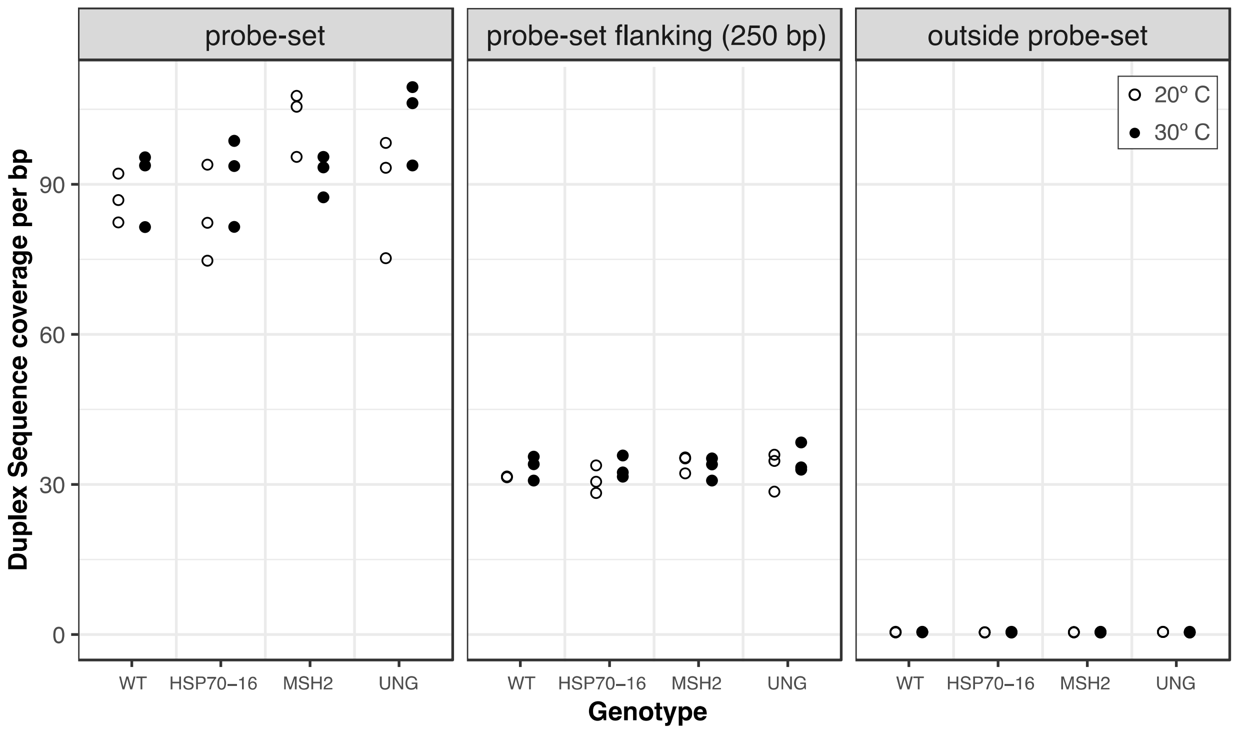

Figure S2. Duplex Sequencing coverage of the probe-set (panel 1), the 250 bps flanking the probe-set (panel 2) and the rest of the genome, outside of the probe-set (panel 3).
