# Supplementary material for: Exploring the Relationship Between Gene Expression and Low-Frequency Somatic Mutations in Arabidopsis with Duplex Sequencing
Source: Genome Biol Evol. 2024 Oct 4;16(10):evae213. doi: 10.1093/gbe/evae213 (PMC11489876; doi:10.1093/gbe/evae213)
Supplement: evae213_Supplementary_Data [file evae213_supplementary_data.zip › athal_mut_waneka_GBE.supp_mat.tables.240907.pdf]

SUPPLEMENTARY TABLES

Table S1. Differentially expressed genes from the RNA-seq analysis identified with DESeq2. The cutoff criteria used to identify genes in the 6 categories of interest (see main text) are listed in the table.

Category	Genotype	Comparison	Corrected p-value	Log fold change	Average normalized cover of each treatment	Number of genes	Included in probe-set	Genes retained after arbor repeat filtering
1	WT	Increased exp. at 30°C	0.05	> 2	Minimum coverage > 5	615	100 with greatest LFC	84
2	WT	Increased exp. at 20°C	0.05	< -2	Minimum coverage > 5	332	100 with lowest LFC	80
3	WT	Constitutive low exp.	0.05	50 genes with LFC closest to 0	50 genes with lowest coverage (ranges from 129 to 400	50	50	44
4	WT	Constitutive high exp.	0.05	50 genes with LFC closest to 0	50 genes with highest coverage (ranges from 8384 to 68053	50	50	45
5	WT vs. HSP70-16	Interaction between genotype and temp	0.05	>2	Minimum coverage > 5	106 (39 of which are also in group 1)	92 with highest LFC	81
6	WT vs HSP70-60	Interaction between genotype and temp	0.05	<-2	Minimum coverage > 5	8 (5 of which are also in group 2)	All 8	5
total							400	339

Table S2. Duplex Sequencing coverage for each replicate

Sample	Mean Depth of Coverage	Total Duplex Seq. Data (bp)
WT 20°C A	86.86	74273348
WT 20°C B	92.16	78809954
WT 20°C C	82.40	70459706
WT 30°C A	81.46	69660673
WT 30°C B	95.39	81571700
WT 30°C C	93.77	80187868
HSP70-16 20°C A	82.31	70384149
HSP70-16 20°C B	74.75	63917524
HSP70-16 20°C C	93.94	80328860
HSP70-16 30°C A	93.65	80085644
HSP70-16 30°C B	81.50	69690981
HSP70-16 30°C C	98.70	84396810
MSH2 20°C A	105.53	90244630
MSH2 20°C B	95.50	81667422
MSH2 20°C C	107.69	92087225
MSH2 30°C A	95.50	81666433
MSH2 30°C B	87.40	74739952
MSH2 30°C C	93.40	79871709
UNG 20°C A	98.30	84059203
UNG 20°C B	93.33	79804898
UNG 20°C C	75.23	64327096
UNG 30°C A	109.44	93588299
UNG 30°C B	93.79	80203757
UNG 30°C C	106.23	90842455

Table S3. Putative fixed SNVs removed before downstream analysis of Duplex Sequencing data

Genotype	Chromosome	Position	Substitution type	Shared among all replicates
ung	2	2016156	AT→GC	yes
wild-type	2	14827204	CG→AT	yes
msh2	4	14827204	CG→AT	yes

Table S4. Putative fixed indels removed before downstream analysis of Duplex Sequencing data

Chrom	Pos	Indel Type	Genotype	Number of Reps (of 6)	Indel Length	Indel Seq
Chrom1	2243387	I	MSH2	6	1	G
Chrom1	2243387	I	WT	6	1	G
Chrom1	2243387	I	UNG	6	1	G
Chrom1	2243387	I	HSP70	6	1	G
Chrom1	2269740	D	MSH2	6	1	A
Chrom1	2270545	D	MSH2	5	1	T
Chrom1	2437835	D	MSH2	5	1	T
Chrom1	5291180	D	MSH2	6	1	T
Chrom1	6591532	I	MSH2	6	1	A
Chrom1	6591532	I	WT	6	1	A
Chrom1	6591532	I	UNG	6	1	A
Chrom1	6591532	I	HSP70	6	1	A
Chrom1	8551177	I	MSH2	6	1	G
Chrom1	8551177	I	WT	6	1	G
Chrom1	8551177	I	UNG	6	1	G
Chrom1	8551177	I	HSP70	6	1	G
Chrom1	11646952	D	MSH2	6	1	T
Chrom1	13533273	I	MSH2	6	3	AGA
Chrom1	13533273	I	WT	6	3	AGA
Chrom1	13533273	I	UNG	6	3	AGA
Chrom1	13533273	I	HSP70	6	3	AGA
Chrom1	17886514	D	MSH2	6	1	A
Chrom1	23734915	D	MSH2	6	1	A
Chrom1	26640491	D	MSH2	6	1	A
Chrom2	11236090	D	MSH2	4	1	A
Chrom2	11567248	I	MSH2	4	1	T
Chrom2	11567248	I	WT	6	1	T
Chrom2	11567248	I	UNG	6	1	T
Chrom2	11567248	I	HSP70	6	1	T
Chrom2	17464171	D	MSH2	6	1	T
Chrom3	4833763	D	MSH2	6	1	A
Chrom3	8412456	D	MSH2	4	1	T
Chrom3	18338647	D	MSH2	6	1	T
Chrom4	13742764	D	MSH2	6	1	T

Chrom4	16470637	I	MSH2	6	1	T
Chrom4	16470637	I	WT	6	1	T
Chrom4	16470637	I	UNG	6	1	T
Chrom4	16470637	I	HSP70	6	1	T
Chrom5	2974730	D	MSH2	4	1	T
Chrom5	7718829	D	MSH2	6	1	T
Chrom5	25010019	D	MSH2	6	1	A

Table S5. Indel mutations in *msh2* mutant lines

Sample	Deletions	Insertions
MSH2 20°C A	44	7
MSH2 20°C B	33	2
MSH2 20°C C	33	5
MSH2 30°C A	47	4
MSH2 30°C B	43	5
MSH2 30°C C	47	6
total	247	29

Table S6. Paired t-test results of category 3 vs category 4 mutation rates in *msh2*$^{-}$ lines (two-tailed)

Temp	Mutation class	Group 3 ave. variant frequency	Group 4 ave. variant frequency	P value
20 °C	SNV	1.02×10^{-07}	1.04×10^{-07}	0.9771
30 °C	SNV	7.25×10^{-08}	9.47×10^{-08}	0.6815
20 °C	INDEL	1.19×10^{-07}	1.38×10^{-07}	0.1615
30 °C	INDEL	1.17×10^{-07}	1.72×10^{-07}	0.0695

Table S7. Mutant lines used, all sourced from ABRC

**Gene**	**AGI**	**Mutant Allele**	**Ref**
HSP70-16	AT1G11660	SALK_028829	(Ran *et al.* 2020)
MSH2	AT3G18524	SALK_002708	(Belfield *et al.* 2018)
UNG	AT3G18630	CS308297	(Cordoba-Canero *et al.* 2010)

Table S8. PCR primers used to identify mutant alleles in the three mutant lines

**Gene/line**	Fwd Primer	Rev Primer
HSP70-16 WT	TACGCACTCACTTGCATTCAC	TGTGTTATCGCAGTTGCAAAG
HSP70-16 Mut	ATTTTGCCGATTTCGGAAC	TGTGTTATCGCAGTTGCAAAG
MSH2 WT	TCACCACGATGATGTCAAGAG	AGGAGCTGTCAAAAGGAGCTC
MSH2 Mut	ATTTTGCCGATTTCGGAAC	AGGAGCTGTCAAAAGGAGCTC
UNG WT	ACTTGGAGAAGGTAAAGCAATTCA	CCATACAAAATATAATACACCACCACTC
UNG Mut	ACTTGGAGAAGGTAAAGCAATTCA	ATATTGACCATCATACTCATTGC

Table S9. Read counts for the 12 RNA-seq libraries

Sample	Count of read pairs
HSP70-16 20°C A	29689895
HSP70-16 20°C B	32052311
HSP70-16 20°C C	33450418
HSP70-16 30°C A	32567642
HSP70-16 30°C B	31456737
HSP70-16 30°C C	29678098
WT 20°C A	30417658
WT 20°C B	54410188
WT 20°C C	42449872
WT 30°C A	34353207
WT 30°C B	36605678
WT 30°C C	37953073

Table S10. Read counts for the 24 Duplex Sequencing libraries

Sample	Count of read-pairs
HSP70-16 20°C A	102214316
HSP70-16 20°C B	88105828
HSP70-16 20°C C	106355604
HSP70-16 30°C A	88061502
HSP70-16 30°C B	99506728
HSP70-16 30°C C	112263590
MSH2 20°C A	106838516
MSH2 20°C B	90724220
MSH2 20°C C	111544972
MSH2 30°C A	115206890
MSH2 30°C B	93741162
MSH2 30°C C	111444292
UNG 20°C A	113380236
UNG 20°C B	110455064
UNG 20°C C	108883106
UNG 30°C A	91537708
UNG 30°C B	87766824
UNG 30°C C	123532620
WT 20°C A	100905496
WT 20°C B	102443086
WT 20°C C	116973524
WT 30°C A	97650342
WT 30°C B	105779540
WT 30°C C	110474398
